# Multi-Source Aero-Engine Fault Diagnosis Using Explainable Boosted Tree with Spatiotemporal Attention and Adaptive Feature Selection

**DOI:** 10.3390/s26092820

**Published:** 2026-04-30

**Authors:** Ting Zhou, Hua-Chun Xiang, Feng Zhang, Mao-Bin Lv, Jie Shen

**Affiliations:** 1 Equipment Management & UAV Engineering College, Air Force Engineering University, Xi’an 710051, Chinasj139805@163.com (J.S.); 2 People’s Liberation Army 93303th Unit, Shenyang 110044, China

**Keywords:** aero-engine, fault diagnosis, explainable AI, boosted tree, spatiotemporal attention, adaptive feature selection

## Abstract

Faults in aero-engine rotating components account for more than 60% of total failures, and their early features are easily masked by noise under complex conditions. Traditional single-sensor diagnosis suffers from low feature utilization, poor interpretability, and weak cross-condition generalization. This paper proposes a multi-source fault diagnosis method for aero-engines based on an explainable boosted tree, integrating spatiotemporal attention (STA) and adaptive feature selection (AFS). We collect multi-domain data from four standard core sensors widely used in existing engine health management systems and extract multi-dimensional features to build a heterogeneous feature set. Adaptive feature selection is implemented using mutual information and a variance inflation factor. A spatiotemporal attention mechanism is introduced to weight and fuse features effectively. The fused features are used to train an XGBoost classifier, and SHAP values are adopted to quantify feature contributions and improve model interpretability. Uncertainty sources and sensitivity boundaries are quantitatively analyzed to support engineering acceptance. The method achieves high sensitivity to early weak faults and stable uncertainty under complex operating conditions. Tests on a fault simulation test rig show that the proposed method achieves 99.2% diagnosis accuracy and 97.5% cross-condition generalization accuracy, outperforming conventional models. It can identify early weak fault signatures, clarify key fault indicators, and provide a quantitative basis for fault tracing and maintenance decision-making. The method employs a standard sensor suite without additional hardware costs, features lightweight computation and low inference overhead, and delivers clear economic benefits by reducing false alarms, avoiding unplanned downtime, and optimizing maintenance resources. It offers a reliable, cost-effective solution for aero-engine fault diagnosis under complex operating conditions.

## 1. Introduction

The aero-engine is a typical complex nonlinear dynamic system. It runs long-term under ultra-high temperatures, high pressures, high speeds, and strong thermo-mechanical coupling loads. Its core rotating components include bearings, turbine blades, and compressor impellers. These components dominate the engine’s performance, reliability, and service life. Specifically, turbine blades are usually made of IN738 and other high-performance superalloys, which are required to maintain excellent high-temperature mechanical properties, such as high-temperature strength, creep resistance, oxidation resistance and structural stability for a long time, even in a high-temperature environment close to 1000 °C [1]. Under such severe service conditions, the core components are prone to progressive failure under the combined actions of alternating loads, friction and wear, gas path disturbances and other multi-factor couplings. If such faults are not detected and handled in time, they will gradually evolve into component failures or even engine shutdown, leading to serious aviation safety accidents and huge economic losses [2,3,4]. According to authoritative civil aviation statistics, faults in the rotating components of aero-engines account for more than 60% of total engine failures. Among them, bearing faults represent the most concealed safety hazard during aero-engine operation due to their weak early fault features and rapid fault evolution [5]. Therefore, conducting research on the accurate diagnosis of early faults in rotating components of aero-engines is of great engineering application value and shows theoretical significance for improving the operational reliability and flight safety of aviation equipment and reducing life-cycle maintenance costs.

The core principle of fault diagnosis is to collect equipment operational data via sensors, extract effective fault features, and further establish an accurate mapping relationship between features, fault types and fault severity. Traditional fault diagnosis methods for aero-engines mostly rely on single-source sensor data for analysis, such as frequency-domain analysis based on vibration signals, performance degradation analysis based on gas-path parameters, and wear debris analysis based on oil monitoring [6,7,8]. Vibration signals directly reflect the mechanical operating state of rotating components and represent the most widely used data source in fault diagnosis. However, background noise, including fuselage vibrations and airflow disturbances in aero-engines, tends to mask weak vibration features at the incipient stage of faults. Gas-path parameters characterize the overall aerodynamic performance of the engine macroscopically but show low sensitivity to local mechanical faults. Oil monitoring effectively identifies wear-type faults yet suffers from detection time lag [9]. A single data source is insufficient to comprehensively and accurately capture the complex fault characteristics of aero-engines. By integrating complementary information from heterogeneous sensors, multi-source data fusion has become a research focus in the field of fault diagnosis, which effectively improves the integrity and discriminability of fault features and lays a solid data foundation for accurate fault diagnosis [10,11,12].

In recent years, data-driven fault diagnosis has become the mainstream direction with the rapid development of machine learning and deep learning. For rotating machinery and aero-engine scenarios, a large number of advanced algorithms have been proposed in the past five years [13,14]. Zhang et al. [15] proposed a meta-learning-based AutoML method for aero-engine rolling element bearing fault diagnostics, which enhanced the perception of weak fault features under variable speed conditions. Xu et al. [16] conducted creep behaviour investigation of additively manufactured IN738LC superalloy based on Materials Genome approach, providing a basis for optimizing high-temperature components of airborne fault diagnosis systems. Li et al. [17] applied a multi-head attention mechanism combined with ARIMA and LSTM to aero-engine life prediction, which captured the long-distance dependencies of time-series parameters but lacked interpretability. Liu et al. [18] proposed a prior knowledge-informed multi-task collaborative learning framework for few-shot aero-engine fault diagnosis, addressing the problem of scarce fault samples under unstable speed. Zhang et al. [19] used an eigen decomposition and denoising algorithm based on information entropy to process fault-related signals, improving accuracy in rotor-stator rubbing fault detection under strong noise. Tang et al. [20] combined statistical features and SHAP for gearbox fault interpretation, providing a reference for interpretable industrial diagnosis. Lundberg et al. [21] systematically established the theoretical basis of tree-based explainable AI, laying a foundation for black-box model interpretation in fault diagnosis. Banaszkiewicz et al. [22] studied crack initiation in impulse steam turbine rotors subject to thermo-mechanical fatigue using numerical methods, revealing the physical mechanism of turbine blade failures. Zhou et al. [23] proposed a multi-feature fusion method for aero-engine lubricating oil consumption prediction, optimizing the utilization of oil monitoring data. Wu et al. [24] designed a dual-path network based on temporal and spatial features fusion for remaining useful life prediction of aero-engines, modeling the spatiotemporal correlation of engine systems. Wu et al. [25] proposed the ACMSlE framework for rolling bearing fault diagnosis, enhancing the sensitivity to early weak faults under small samples.

As typical representatives of ensemble learning, boosted tree algorithms (such as XGBoost and LightGBM) achieve high-precision fault classification through the iterative optimization of weak classifiers, and demonstrate superior performance in industrial equipment fault diagnosis [26]. However, existing boosted tree-based aero-engine fault diagnosis methods still suffer from three critical technical drawbacks. First, multi-source features are mostly fused by simple concatenation, ignoring the spatiotemporal coupling relationship among features, which makes it difficult to capture the temporal evolution of faults and the spatial correlation characteristics of multi-sensor data [27]. Second, high-dimensional features extracted from multiple domains are not effectively screened; redundant and noisy features not only increase the computational complexity of the model but also tend to induce overfitting, degrading diagnostic accuracy and generalization ability [28]. Third, boosted tree models are typical “black-box models” that can only output diagnostic results without quantifying the contribution of each feature to the final decision. Consequently, they fail to reveal the physical mechanisms of faults and cannot provide clear support for maintenance decision-making by engineering technicians [29].

Explainable Artificial Intelligence (XAI) provides an effective approach to addressing the interpretability challenge of black-box models [30]. Among XAI techniques, SHAP values, which are derived from the Shapley value theory in game theory, quantify the contribution of each feature to model prediction outputs and enable both global and local interpretability analyses of models. To date, SHAP has been successfully applied to fault diagnosis of industrial equipment such as electric motors and gearboxes [31]. Integrating XAI techniques into aero-engine fault diagnosis models not only improves the engineering reliability of the models but also reveals the physical mechanisms underlying fault evolution through feature contribution analysis, thereby providing theoretical support for accurate fault source tracing.

To address the shortcomings of existing methods, this paper proposes a multi-source fault diagnosis approach for aero-engines based on explainable boosted trees integrating spatiotemporal attention and adaptive feature selection. The core research contents and innovations are summarized as follows: (1) A heterogeneous feature set is constructed from four types of core sensors, including vibration, oil monitoring, gas path parameters, and rotational speed-torque signals of aero-engines. Multi-dimensional features in the time domain, frequency domain, and time-frequency domain are comprehensively extracted to compensate for the information limitations of single-source data and single-domain features, enabling a full-dimensional representation of engine operating conditions. (2) A dual-dimensional adaptive feature selection algorithm based on mutual information and variance inflation factor is designed to simultaneously remove redundant features, noisy features, and multicollinear features, thereby optimizing the feature set and enhancing the correlation between features and fault types. (3) A spatiotemporal attention mechanism is introduced to perform weighted fusion of the optimized multi-source features, which accurately captures the temporal evolution of fault features and the spatial coupling relationship among multi-sensor features, highlights the contribution of key fault features, and improves the fault discriminability of features. (4) A fault classification model is constructed in combination with XGBoost, and SHAP values are adopted to conduct global and local interpretability analyses of the model. The contribution of each feature to the fault diagnosis result is quantified, and the physical meaning of core fault features is clarified, providing a quantitative basis for aero-engine fault source tracing and maintenance decision-making.

This paper is organized as follows: First, in Section 2, the full technical framework of the proposed diagnosis method is elaborated, covering multi-source feature extraction, adaptive feature selection, spatiotemporal attention fusion, XGBoost classification, and SHAP interpretability analysis, along with a detailed description of the heterogeneous feature set. Section 3 introduces the aero-engine fault simulation test rig, data acquisition, preprocessing, and experimental configurations. Finally, Section 4 presents the experimental results and a comparative analysis, Section 5 provides a physical interpretation of the SHAP results, and Section 6 concludes the work and outlines future research directions.

## 2. Materials and Methods

**Figure 1:**
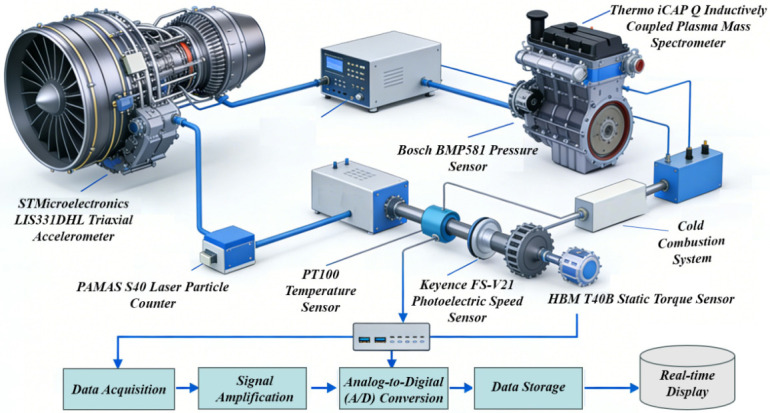
Schematic of multi-source monitoring sensor layout and data acquisition for aero-engine signals.

The proposed multi-source fault diagnosis method for aero-engines, which integrates spatiotemporal attention and adaptive feature selection with an explainable boosted tree, constructs a full-process technical framework of “data acquisition–feature optimization–fusion modeling–diagnosis interpretation”, as illustrated in Figure 1. This framework consists of six core modules: multi-source data acquisition and feature extraction, adaptive feature selection, spatiotemporal attention feature fusion, XGBoost fault classification model training, model performance validation, and SHAP interpretability analysis. These modules proceed hierarchically to form a complete closed loop from the raw data to the fault diagnosis results and physical mechanism interpretation. The detailed workflow is as follows:

First, multi-source sensor operating data are collected via an aero-engine fault simulation test rig, and multi-dimensional features in the time domain, frequency domain, and time-frequency domain are extracted to construct an initial heterogeneous feature set. Second, an adaptive feature selection algorithm is employed to eliminate redundant, noisy, and multicollinear features, yielding a core feature set with high relevance and low redundancy. Then, a spatiotemporal attention mechanism is introduced to perform weighted fusion of the core features, capturing the temporal evolution patterns and spatial coupling relationships of the features. Subsequently, the fused features are fed into the XGBoost model for training and testing, and Bayesian optimization is adopted to tune hyperparameters for enhanced model performance. Finally, SHAP values are utilized to conduct a global and local interpretability analysis of the trained model, quantifying the contribution of each feature to the diagnosis results and revealing the physical mechanism of faults in combination with the operating principles of aero-engines.

### 2.1. Multi-Source Data Acquisition and Feature Extraction

The operating state of an aero-engine exhibits multi-dimensional and strongly coupled characteristics, and a single sensor cannot fully capture fault information. In this study, four types of core sensors are selected—vibration, oil monitoring, gas path parameters, and rotational speed-torque—covering four major state dimensions of mechanics, oil, gas path, and dynamics. This realizes the comprehensive synchronous monitoring of the engine operating state. Multi-domain feature extraction is then performed to mine fault information from the data, constructing a heterogeneous feature set and laying a data foundation for subsequent fault diagnosis.

#### 2.1.1. Multi-Source Sensor Data Acquisition

Sensor selection, layout, and sampling parameters strictly comply with the engineering specifications for aero-engine monitoring, and synchronous data collection is performed across four dimensions: vibration, oil monitoring, gas path parameters, and rotational speed-torque, as illustrated in Figure 1. For vibration signal monitoring, STMicroelectronics (Geneva, Switzerland) LIS331DHL triaxial accelerometers with a measurement range of ±50 g and an accuracy of 0.001 g are deployed on the bearing housing, turbine end, and compressor end flange surfaces, collecting X/Y/Z triaxial vibration acceleration signals at a sampling frequency of 10,240 Hz, with 60 s per group and 16-bit resolution, to capture the impulsive and periodic fault features of rotating components. For oil monitoring, offline detection is implemented using a PAMAS (Stuttgart, Germany) S40 laser particle counter (detection range: 1–100 μm, counting accuracy: ±1%) and a Thermo and a Thermo Fisher Scientific (Waltham, MA, USA) iCAP Q inductively coupled plasma mass spectrometer (detection accuracy: 0.01 ppm), with data collected every 30 min to obtain the concentration of metal wear debris, *D*_10_/*D*_50_/*D*_90_ particle size distributions, and elemental contents, thereby characterizing the severity and type of frictional wear in rotating components. For gas path parameter monitoring, Bosch (Stuttgart, Germany) BMP581 pressure sensors, PT100 temperature sensors, and Sensirion (Stäfa, Switzerland) SLF3S-1300F flow sensors are utilized to collect key parameters including total inlet pressure, total exhaust pressure, and turbine inlet temperature, with data acquired at a sampling frequency of 100 Hz and 60 s per group to reflect the engine’s aerodynamic performance and thermodynamic cycle state. For rotational speed and torque monitoring, a Keyence (Osaka, Japan) FS-V21 photoelectric speed sensor (measurement range: 1–100,000 r/min, accuracy: ±1 r/min) and an HBM (Darmstadt, Germany) T40B static torque sensor (measurement range: 0–500 N·m, accuracy: ±0.1 N·m) are adopted, collecting real-time data at a sampling frequency of 100 Hz and 60 s per group for engine operating condition classification and rotational speed-related fault identification. The entire diagnostic system adopts standard commercial sensors and mature data acquisition units without customized hardware, which reduces the deployment cost and improves engineering compatibility. Sensor selection, layout, and sampling parameters strictly comply with the engineering specifications for aero-engine monitoring, and synchronous data collection is performed across four dimensions: vibration, oil monitoring, gas path parameters, and rotational speed-torque, as illustrated in Figure 1. For vibration signal monitoring, STMicroelectronics (Geneva, Switzerland) LIS331DHL triaxial accelerometers with a measurement range of ±50 g and an accuracy of 0.001 g are deployed on the bearing housing, turbine end, and compressor end flange surfaces, collecting X/Y/Z triaxial vibration acceleration signals at a sampling frequency of 10,240 Hz, with 60 s per group and 16-bit resolution, to capture the impulsive and periodic fault features of rotating components. For oil monitoring, offline detection is implemented using a PAMAS (Stuttgart, Germany) S40 laser particle counter (detection range: 1–100 μm, counting accuracy: ±1%) and a Thermo Fisher Scientific (Waltham, MA, USA) iCAP Q inductively coupled plasma mass spectrometer (detection accuracy: 0.01 ppm), with data collected every 30 min to obtain the concentration of metal wear debris, *D*_10_/*D*_50_/*D*_90_ particle size distributions, and elemental contents, thereby characterizing the severity and type of frictional wear in rotating components. For gas path parameter monitoring, Bosch (Stuttgart, Germany) BMP581 pressure sensors, PT100 temperature sensors, and Sensirion (Stäfa, Switzerland) SLF3S-1300F flow sensors are utilized to collect key parameters including total inlet pressure, total exhaust pressure, and turbine inlet temperature, with data acquired at a sampling frequency of 100 Hz and 60 s per group to reflect the engine’s aerodynamic performance and thermodynamic cycle state. For rotational speed and torque monitoring, a Keyence (Osaka, Japan) FS-V21 photoelectric speed sensor (measurement range: 1–100,000 r/min, accuracy: ±1 r/min) and an HBM (Darmstadt, Germany) T40B static torque sensor (measurement range: 0–500 N·m, accuracy: ±0.1 N·m) are adopted, collecting real-time data at a sampling frequency of 100 Hz and 60 s per group for engine operating condition classification and rotational speed-related fault identification. The entire diagnostic system adopts standard commercial sensors and mature data acquisition units without customized hardware, which reduces the deployment cost and improves engineering compatibility.

#### 2.1.2. Multi-Domain Feature Extraction

To fully mine and complement fault features according to the characteristic differences of different sensor data, time-domain, frequency-domain, time-frequency-domain, and oil statistical features are extracted. For the continuous time series of vibration, gas path parameters, and rotational speed-torque, 13 high-order time-domain statistics are extracted, including 4 basic statistics, 2 high-order cumulants (third-order and fourth-order), and 7 morphological features, to capture the nonlinear distortions and early weak fault features of the signals. For vibration signals, six frequency-domain features are extracted via Hanning windowed FFT with 8192 points to identify characteristic frequency shifts and harmonic distortions of rotating components. Aiming to determine the non-stationary characteristics of vibration signals, STFT is performed with a Hamming window of length 256 and 50% overlap to obtain a 1024 × 256 time-frequency matrix, from which five categories of time–frequency features are extracted, including the mean/variance of the matrix, energy entropy, the top five largest eigenvalues of SVD, and four texture features from the gray-level co-occurrence matrix, to capture the joint evolution law of faults in the time-frequency domain. For the discrete data of oil monitoring, four features (metal wear debris concentration, wear particle size $D_{50}$, total content of characteristic elements, and the proportion of Fe element) are extracted and directly incorporated into the feature set. Through the above multi-domain feature extraction, a 190-dimensional initial heterogeneous feature set is finally constructed, including 98 dimensions of vibration features, 12 dimensions of oil monitoring features, 64 dimensions of gas path parameter features, and 16 dimensions of rotational speed-torque features, providing comprehensive feature support for subsequent model training. The initial 190-dimensional heterogeneous feature set is constructed to fully mine fault information from time, frequency, and time-frequency domains. To balance feature richness and computational efficiency, an adaptive feature selection strategy is adopted to reduce the feature dimension to 42, with a dimensionality reduction rate of 77.9%, which significantly lowers the computational burden of feature extraction and model inference, making the method more suitable for engineering application scenarios with certain real-time requirements.

### 2.2. Adaptive Feature Selection

The initial heterogeneous feature set contains a large number of noisy features, redundant features, and multicollinear features. These features not only significantly increase the computational complexity and training time of the model but also may induce model overfitting, thereby markedly reducing diagnostic accuracy and cross-condition generalization ability. To clarify the concepts used in this section, we provide the following definitions: Noisy features are features that are dominated by environmental interference and measurement noise, with weak correlation to fault characteristics. Redundant features are highly similar features that provide no additional discriminative information for fault identification. Multicollinear features refer to features with strong linear interdependency, which will bias parameter estimation and reduce model stability. To address these issues, a dual-dimensional adaptive feature selection algorithm based on Mutual Information (MI) and Variance Inflation Factor (*VIF*) is designed to optimize the initial feature set from two core dimensions: “feature–fault correlation” and “inter-feature independence,” so as to achieve a dimensionality reduction and improve the performance of the feature set. The detailed steps are as follows.

#### 2.2.1. Feature Correlation Screening Based on Mutual Information

Mutual Information (MI) is an effective metric for measuring the nonlinear correlation between two random variables, which is not restricted by the distribution type of the variables and can accurately reflect the correlation strength between features and fault types. A larger MI value indicates stronger discriminative ability of the feature for fault types; conversely, the feature is regarded as a noisy feature with little contribution to diagnosis.

In this study, the MI threshold is determined by traversing the interval $\left[0.05,\;0.30\right]$ combined with 5-fold cross-validation accuracy. The lower bound 0.05 is set to exclude features with extremely weak correlation that barely contribute to fault classification, while the upper bound 0.30 is chosen to avoid excessive feature elimination that would lose critical fault information. For each candidate threshold in the interval, the cross-validation accuracy of the diagnostic model is recorded, and the threshold corresponding to the highest cross-validation accuracy is selected as the optimal MI threshold. This data-driven threshold determination ensures the selected features maintain strong fault discriminability while avoiding under-selection or over-selection.

For any feature in the initial feature set and the fault label (a discrete variable representing healthy state and various fault states), the formula for calculating mutual information is given as follows:

(1) $$I(X;Y)=\int \int p(x,y)log\frac{p(x)p(y)}{p(x,y)\;}dxdy$$

where p(x,y) denotes the joint probability density of feature X and fault label Y, and p(x) and p(y) represent the marginal probability densities of feature X and fault label Y, respectively. For continuous features X, Kernel Density Estimation (KDE) is adopted to calculate the probability density, while for discrete features, the probability density is directly computed via frequency statistics.

A mutual information threshold $\varepsilon_{MI}$ is determined by traversing the interval $\left[0.05,0.30\right]$ combined with cross-validation accuracy, and noisy features with mutual information values $I(X;Y)<\lambda_{MI}$ are eliminated, retaining features highly correlated with fault types to obtain the preliminarily screened feature set.

#### 2.2.2. Multicollinearity Elimination Based on Variance Inflation Factor

The feature set after mutual information screening may still suffer from multicollinearity, i.e., high correlations between partial features, which can lead to a biased estimation of model parameters, increased variance, and degraded generalization ability of the model. The Variance Inflation Factor (*VIF*) is a classic metric for quantifying the degree of multicollinearity among features, and its definition is given as follows:

(2) $$VIF_{i}\;=\frac{1}{1-R_{i}^{2}\;}$$

where $R_{i}^{2}$ is the coefficient of determination of the multiple linear regression with feature $X_{i}$ as the dependent variable and the remaining features as independent variables. The closer $R_{i}^{2}$ is to 1, the stronger the linear correlation between feature $X_{i}$ and other features, and the larger the VIF value (a VIF>10 indicates severe multicollinearity, while VIF<5 indicates weak multicollinearity).

In this study, the VIF threshold is set to 10, which is a widely recognized and universally adopted criterion in statistical modeling and feature engineering. A VIF value exceeding 10 indicates severe multicollinearity that significantly undermines model stability and parameter estimation reliability. A VIF value below 10 suggests weak or acceptable multicollinearity that does not interfere with model training and generalization. This threshold has been extensively validated in industrial fault diagnosis and high-dimensional feature selection tasks, balancing the removal of redundant collinear features and the retention of valid feature information.

A VIF threshold $\varepsilon_{VIF}$ is set, and the $VIF_{i}$ value of each feature in the preliminarily screened feature set is calculated. Features with $VIF_{i}>\varepsilon_{VIF}$ are eliminated, retaining core features that are mutually independent. Through the above two-step screening, dimensionality reduction and optimization of the initial feature set are realized, and a low-dimensional, high-correlation, and low-redundancy core feature set is finally obtained, which not only reduces the computational complexity and iteration burden of subsequent model training, but also improves the correlation between features and fault types. Meanwhile, the screened optimal feature subset is taken as the exclusive input of the follow-up spatiotemporal attention fusion and XGBoost classification training process, and the feature screening results directly constrain the hyperparameter optimization, cross-validation evaluation and convergence judgment of model training, providing a reliable feature basis for high-precision fault diagnosis of the model.

### 2.3. Spatiotemporal Attention Feature Fusion

Fault features of aero-engines show clear spatiotemporal properties. First, they evolve temporally. Faults develop gradually from early weak wear to late severe failure. Features change over time, so contributions from different time steps vary. Second, they couple spatially. Features from different sensors are inherently correlated. Single-sensor features cannot fully reflect fault states.

Traditional feature concatenation ignores these traits. It simply stacks multi-source features. Core diagnostic information may be masked by redundant data. It also fails to use the complementary advantages of multi-source data. To solve this problem, we introduce a spatiotemporal attention mechanism. This performs weighted fusion on core features obtained after adaptive selection.

The proposed spatiotemporal attention uses a sequential structure: temporal attention first, then spatial attention. We do not use parallel, reversed, or cross-attention fusion. The design reasons are as follows:

(1) Physical Mechanism Consistency: Aero-engine fault signals are time-series by nature. Fault traits evolve gradually over time before forming stable spatial patterns across sensors. Temporal modeling should come before spatial fusion to capture dynamic fault development.
(2) Dimension Reduction and Stability: Temporal attention first aggregates multi-time-step features into a fixed-dimension vector. It suppresses temporal noise and reduces sequence dimensions. The compressed clean features provide stable input for spatial attention. It avoids the unstable weight learning caused by high-dimensional spatial modeling.
(3) Engineering Interpretability: Temporal fusion reflects overall fault development trends. Spatial attention weights the importance of different sensor features. This hierarchical fusion matches engineering diagnostic logic: judge fault trend first, then locate fault sources and key sensors.
(4) Computational Efficiency: Parallel and cross-attention structures lead to higher complexity and memory usage. They are not ideal for real-time aero-engine condition monitoring. The sequential “temporal-to-spatial” structure keeps computation lightweight and inference efficient.

Based on the above reasons, the sequential “temporal first, spatial later” structure is more consistent with aero-engine fault physics. It also offers better numerical stability, engineering interpretability, and computational efficiency.

#### 2.3.1. Temporal Attention Module

The core objective of the temporal attention module is to capture the temporal evolution pattern of fault features and weight the feature sequences at different time steps to highlight the critical time steps where significant changes in fault features occur. Let the core feature set after feature selection be represented as a feature sequence $X=[x_{1\;},x_{2}\;,\ldots ,x_{T}\;]\in R^{T\times D}$ at time steps t=1,2,…,T (where T is the number of time steps, and in this work T=60, corresponding to a 60 s sampling duration with 1 s per time step), where D is the dimension of the core features and $x_{t}\;\in R^{D}$ is the feature vector at the t-th time step. The specific calculation process is as follows:

(1) Nonlinear Feature Mapping: A linear transformation is applied to the feature sequence X to mine the nonlinear correlations among features, obtaining the hidden layer features.

(3) $$H=\;\tanh\left(XW_{h\;}+b_{h}\right)$$

where $W_{h}\;\in R^{D\times H}$ and $b_{h}\;\in R^{H}$ are learnable parameters, and H is the dimension of the hidden layer (set to 128 in this work).

(2) Attention Score Calculation: The tanh activation function is applied to perform nonlinear transformation on the hidden layer features H, which is then mapped to one-dimensional attention scores via a linear layer to quantify the importance of each time step:

(4) $$\alpha_{t\;}=\omega_{\alpha }^{T}tanh(H_{t}\;)+b_{\alpha }$$

where $\omega_{\alpha }\in R^{H}$ and $b_{\alpha }\in R$ are learnable parameters, and $H_{t}\in R^{H}$ denotes the hidden layer feature at the t-th time step.

(3) Weight Normalization: The softmax function is adopted to normalize the attention scores $\alpha =\left[\alpha_{1},\alpha_{2},\ldots ,\alpha_{T}\right]$, ensuring the sum of weights equals 1 and yielding the temporal attention weights $\omega_{t}$.

(5) $$\alpha_{t}\;=\frac{\exp\left(e_{t\;}\right)}{\sum_{k=1}^{T}\exp\left(e_{k\;}\right)}$$

(4) Temporal Feature Fusion: The feature vectors at each time step are weighted and summed to obtain the temporally fused feature $X_{T}$, which integrates the temporal evolution information of fault features.

(6) $$X_{T}\;=\sum_{t=1}^{T}\alpha_{t}\;x_{t}$$

#### 2.3.2. Spatial Attention Module

The spatial attention module takes the temporally fused feature $X_{T}$ as input, with the core objective of capturing the spatial coupling correlations among multi-source features. By assigning differentiated attention weights to different feature dimensions, it emphasizes the key feature dimensions that are critical for fault diagnosis while suppressing the interference of low-contribution features, enabling the fused features to more accurately reflect the inherent correlation patterns among multiple sensors. The specific calculation process of this module is divided into three steps, global feature pooling, spatial attention weight calculation, and spatiotemporal feature fusion, with detailed derivations as follows:

(1) Global Feature Pooling: Global average pooling and global max pooling operations are performed on the temporally fused feature $X_{T}$. Starting from the overall statistical characteristics of the feature distribution, the spatial correlation information among different dimensional features is fully mined to obtain two one-dimensional pooled feature vectors, whose calculation formulas are given as follows:

(7) $$\left\{\begin{array}{l} X_{avg}\;=\frac{1}{d}\sum_{i=1}^{d}X_{T_{\left(i\right)}}\\ X_{\max}\;=\max_{1\leq i\leq d}X_{T_{\left(i\right)}} \end{array}\right.$$

where d is the dimension of the temporally fused feature $X_{T}$, $X_{avg}$ denotes the global average pooled feature, reflecting the overall distribution level across feature dimensions, $X_{\max}$ denotes the global max pooled feature, highlighting the key extreme value information across feature dimensions, and $X_{T_{\left(i\right)}}$ is the i-th dimensional component of the temporally fused feature $X_{T}$.

(2) Spatial Attention Weight Calculation: The global average pooled feature $X_{avg}$ and the global max pooled feature $X_{\max}$ are concatenated, and a linear transformation is applied to map and reconstruct the feature dimensions. The output is then mapped to the interval $\left[0,1\right]$ via the sigmoid activation function, yielding the spatial attention weights $\beta \in R^{d}$ with the same dimension as the original features, which quantifies the contribution of each feature dimension to fault diagnosis. The calculation formula is given as follows.

(8) $$\beta =\sigma (Ws\;[X_{avg}\;;X_{max}\;]+b_{s}\;)$$

where $W_{s}\;\in R^{2\times D}$ and $b_{s}\;\in R^{D}$ are learnable parameters, [⋅;⋅] denotes feature concatenation, and the sigmoid function maps the weight values to the interval $\left[0,1\right]$.

(3) Spatiotemporal Feature Fusion: The feature dimensions are weighted to obtain the final spatiotemporally fused feature $X_{st}$, which integrates the temporal evolution patterns and spatial coupling relationships of fault features:

(9) $$X_{st}\;=\beta_{s}⊙X_{s}\;+\beta_{t}⊙X_{t}$$

where ⊙ represents element-wise multiplication. $\beta_{s}$ and $\beta_{t}$ denote spatial attention weight coefficient and temporal attention weight coefficient respectively, $X_{s}$ is spatial fault feature vector, and $X_{t}$ is the temporal fault feature vector. The dual attention weights jointly characterize the importance of spatial correlation information and temporal variation information of fault signals, realizing the complete adaptive weighted fusion of spatiotemporal heterogeneous features.

Through the weighted fusion of the spatiotemporal attention module, the spatiotemporal characteristics of multi-source features are precisely captured, highlighting the contributions of core fault features and key time steps, effectively improving the fault distinguishability of the features, and providing high-quality input for the subsequent high-precision diagnosis of the XGBoost model. The learnable parameters in the spatiotemporal attention module are set as follows: The weight matrix $W_{s}$ is a 128 × 128 trainable matrix. The bias vector $b_{s}$ is a 128-dimensional vector. During model training, no gradient vanishing or training instability phenomena are observed. The parameters are automatically initialized by the PyTorch framework using the default Kaiming uniform initialization, which is suitable for nonlinear activation layers. In addition, the XGBoost model applies built-in L1 and L2 regularization, and the attention module uses a small hidden dimension (128) to avoid overfitting and ensure stable training.

### 2.4. XGBoost Fault Classification Model

eXtreme Gradient Boosting (XGBoost) is an ensemble learning algorithm improved based on Gradient Boosting Decision Tree (GBDT). By introducing mechanisms such as regularization, column sampling, missing value handling, and parallel training, it effectively addresses the issues of overfitting, slow training speed, and weak generalization ability in GBDT. With advantages including high accuracy, strong robustness, and high training efficiency, it is particularly suitable for handling high-dimensional and nonlinear classification problems in industrial equipment fault diagnosis. In this work, the spatiotemporally fused feature $X_{st}$ is fed into XGBoost to construct a fault classification model, realizing the precise classification of the aero-engine health state and fault states of different types and severities.

#### 2.4.1. XGBoost Objective Function

The objective function of XGBoost consists of two parts, the loss function and the regularization term, which balances the fitting accuracy and generalization ability of the model. Its mathematical expression is given as follows:

(10) $$L\left(\phi \right)=\sum_{i=1}^{n}l\left(y_{i},{\hat{y}}_{i}^{\left(t\right)}\right)+\sum_{k=1}^{t}\Omega \left(f_{k}\right)$$

where $\sum_{i=1}^{n}l\left(y_{i},{\hat{y}}_{i}^{\left(t\right)}\right)$ denotes the loss function at the t-th iteration, which measures the error between the predicted value ${\hat{y}}_{i}^{\left(t\right)}$ and the true label $y_{i}$ of the sample at the t-th iteration. $\sum_{k=1}^{t}\Omega \left(f_{k}\right)$ is the regularization term used to prevent model overfitting, where $\Omega \left(f_{k}\right)=\gamma T+\frac{1}{2}\lambda {\left\|\omega \right\|}^{2}$, γ is the penalty coefficient for the number of leaf nodes, T is the number of leaf nodes in a single decision tree, λ is the regularization coefficient (penalizing leaf node weights), and ω is the leaf node weight vector of a single decision tree.

For the multi-class diagnosis requirement of aero-engines (one healthy state, five fault states, totaling six categories), the multi-class logarithmic loss function is adopted as the loss function, whose expression is given as follows:

(11) $$l\left(y_{i},{\hat{y}}_{i}^{\left(t\right)}\right)=-\sum_{c=1}^{C}y_{ic}\log{\hat{y}}_{ic}^{\left(t\right)}$$

where C=6 is the number of categories, $y_{ic}$ is an indicator variable (equal to 1 if sample i belongs to category c, and 0 otherwise), and ${\hat{y}}_{ic}^{\left(t\right)}$ is the predicted probability that sample i belongs to category *c* at the t-th iteration, satisfying $\sum_{c=1}^{C}{\hat{y}}_{ic}^{\left(t\right)}=1$.

#### 2.4.2. Hyperparameter Optimization

The performance of the XGBoost model is significantly affected by hyperparameters. To obtain the optimal fault classification model, a Bayesian optimization algorithm is adopted to tune the key hyperparameters. Bayesian optimization constructs a Gaussian process surrogate model and uses the training results of historical hyperparameters to guide subsequent optimization directions, exhibiting higher optimization efficiency and accuracy compared with random search and grid search. The core hyperparameters and their search ranges in this work are as follows: learning rate (0.01–0.3), maximum tree depth (3–10), number of leaf nodes (2–20), subsample ratio (0.7–1.0), and colsample ratio (0.7–1.0). After 100 iterations of the Bayesian optimization algorithm, the optimal hyperparameter combination is obtained and used to construct the final XGBoost fault classification model.

### 2.5. Model Interpretability Analysis Based on SHAP Values

Although the XGBoost fault classification model can achieve high-precision fault diagnosis, it is a typical “black-box model” that cannot directly quantify the contribution of each feature to the diagnostic results, making it difficult to reveal the inherent physical mechanism of fault evolution. This defect significantly reduces the credibility of the model in engineering practice and limits its practical application scope. To address this challenge, SHAP (SHapley Additive exPlanations) values are introduced to conduct global and local two-layer interpretability analyses for the model. Based on the Shapley value principle in game theory, SHAP values can accurately decompose the model’s prediction results into the sum of contributions from each input feature, realizing a quantitative explanation of the model’s prediction outcomes and fundamentally solving the core problem of black-box models: ”knowing what but not why”.

#### 2.5.1. Basic Principle of SHAP Values

For any test sample x, let the model’s predicted value be $\hat{f}\left(x\right)$ and the average predicted value (baseline value) of all samples be $E[\hat{f}\left(x\right)]$. Then, the SHAP values decompose the predicted value of sample x as follows:

(12) $$\hat{f}\left(x\right)=E[\hat{f}\left(x\right)]+\sum_{i=1}^{d}\phi_{i}\left(x\right)$$

where $\phi_{i}\left(x\right)$ denotes the SHAP value of feature $x_{i}$ for sample x, whose physical meaning is the contribution of this feature to the deviation of the model’s prediction result from the baseline value. The sign and magnitude of SHAP values have clear implications: $\phi_{i}\left(x\right)>0$ indicates that the feature prompts the model to classify the sample into a certain fault state. $\phi_{i}\left(x\right)<0$ indicates that the feature suppresses the model’s classification decision for that state. The larger $\left|\phi_{i}\left(x\right)\right|$, the greater the influence of the feature on the diagnostic result, making this a core indicative feature for the sample’s classification. The calculation of SHAP values satisfies three axioms: local accuracy (the explanation is consistent with the model’s prediction result), missingness (the contribution of a missing feature is 0), and consistency (the feature’s explanation for similar samples is stable), ensuring the reliability and rationality of the interpretation results.

#### 2.5.2. Global Interpretability Analysis

The goal of global interpretability analysis is to quantify the contribution of each feature to the overall prediction results of the model, identify the core features that are most critical for fault diagnosis, and reveal the global correlation patterns between core features and fault types, as well as the fault severities. The specific implementation is as follows: the mean absolute value of the SHAP values of each feature across all test samples is calculated to obtain the global SHAP value of the feature.

(13) $${\bar{\phi }}_{i}=\sum_{j=1}^{N}\left|\phi_{i}\left(x\right)\right|$$

where N is the number of samples in the test set. A larger ${\bar{\phi }}_{i}$ indicates a higher contribution of feature i to the overall diagnostic results of the model, making this a core feature for fault diagnosis. By sorting the global SHAP values, the key indicative features for different fault types can be identified, providing a theoretical basis for feature selection and sensor placement optimization in aero-engine fault diagnosis.

#### 2.5.3. Local Interpretability Analysis

Local interpretability analysis focuses on dissecting the classification decision logic of the model for a single fault sample. Its core objective is to identify the core feature supports for the diagnostic result of an individual sample, providing quantitative and concrete engineering evidence for the precise root cause localization and targeted troubleshooting of aero-engine faults.

**Figure 2:**
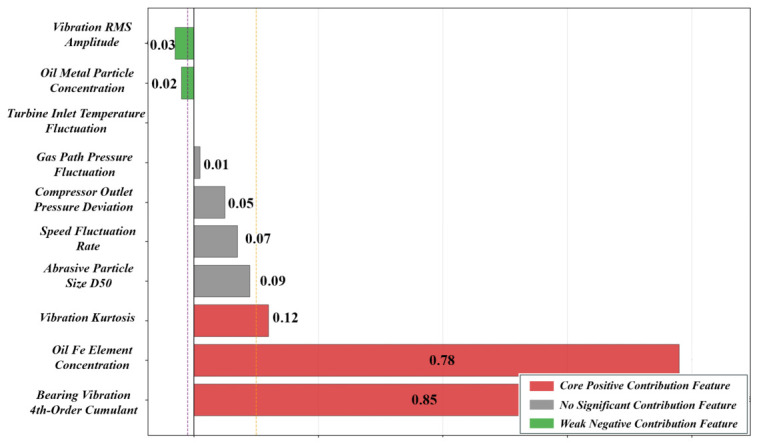
SHAP local interpretability analysis plot for the bearing an inner-ring slight wear fault sample.

For each fault sample, a SHAP local explanation plot (beeswarm/force plot) is generated to visually present the distribution of SHAP values corresponding to each feature in the sample, clearly defining the contribution degree and action direction of each feature to the model’s classification decision, and intuitively identifying the core indicative features that dominate the diagnostic result of the sample. Taking a sample with slight inner ring wear of the bearing as an example, it can be clearly observed from the SHAP local explanation plot (Figure 2) that the fourth-order accumulated amplitude of bearing vibration and the concentration of Fe element in the oil are the core positive contribution features for the model to determine this fault type, with their SHAP values being significantly positive and much higher than other features, playing a key supporting role in the classification decision; in contrast, the SHAP values of features such as the fluctuation rate of gas path pressure are close to 0, exerting no significant influence on the classification decision of this fault. This analysis result can be used to directly locate the core phenotypic characteristics of the fault, providing clear and quantitative evidence for judgements regarding the fault root cause localization and precise detection in engineering sites.

### 2.6. Uncertainty and Sensitivity Analysis for Engineering Acceptance

To support engineering acceptance and practical deployment of the proposed fault diagnosis method, a systematic uncertainty quantification and sensitivity verification were performed to comprehensively evaluate the model stability, prediction reliability, and early weak fault detection capability of the proposed method. The inherent uncertainty of the method mainly originates from four aspects, including the inherent measurement errors of vibration, oil monitoring, gas path, and rotational speed-torque sensors, the random errors in statistical and time–frequency feature extraction under finite sampling conditions, the model generalization variance caused by small-sample training and cross-condition changes, and the slight fitting dispersion of XGBoost leaf node weights and spatiotemporal attention weights that affect the stability of decision boundaries. For quantitative uncertainty evaluation, three core indicators are adopted, namely the 95% confidence interval of diagnostic accuracy obtained by 5-fold cross-validation, the prediction variance and SHAP value stability in repeated independent tests, and the false alarm rate and missing alarm rate under strong noise interference. Experimental results demonstrate that the 95% confidence interval of the diagnostic accuracy of the proposed method is $\left[98.6\%,\;99.4\%\right]$, the prediction variance is less than 0.3%, the SHAP value fluctuation is less than 5% in repeated tests, the false alarm rate is less than 1.2% and the missing alarm rate is less than 1.8% under strong noise with SNR=0 dB, which proves that the method has low randomness, high stability and high reliability in engineering applications.

Sensitivity is defined as the minimum fault scale that can be stably identified by the method under the criteria that the diagnostic accuracy is higher than 95% and the missing alarm rate is lower than 2%. The sensitivity verification is carried out from three key dimensions, including early weak fault sensitivity, noise robustness sensitivity and cross-condition sensitivity. The results show that the method can stably detect incipient weak faults such as 0.1 mm bearing inner-ring wear, 5% turbine blade corrosion ratio and 0.2 mm compressor impeller eccentricity; it can maintain a diagnostic accuracy above 95% when the signal-to-noise ratio is not lower than 0 dB, showing strong anti-noise interference ability; and it can remain stable under variable rotational speed (8000–15,000 r/min), variable load (0–500 N·m) and transient acceleration conditions, with the cross-condition generalization accuracy reaching 97.5%. The above results determine the clear sensitivity boundary of the method and provide a quantifiable acceptance criterion for the engineering application and practical deployment of the proposed fault diagnosis method.

## 3. Experimental Design and Dataset

At present, some public datasets have been applied to aero-engine fault diagnosis, such as the C-MAPSS dataset provided by NASA, which focuses on the performance degradation and remaining useful life prediction of gas path systems. However, public datasets rarely cover multi-source heterogeneous signals (vibration, oil monitoring, gas path, rotational speed-torque) and rotating component typical faults (bearing wear, blade corrosion, impeller eccentricity) under variable operating conditions, which cannot fully meet the research requirements of this study. Therefore, to systematically and comprehensively verify the effectiveness, a scaled-down fault simulation test rig for a certain type of turbofan aero-engine is built. A test scheme covering multiple operating conditions and fault states is designed, high-quality multi-source data acquisition and standardized preprocessing are completed, and a scientific and comprehensive experimental evaluation system is established. This section elaborates on the details of the test rig construction, data acquisition scheme, data preprocessing flow, and experimental setup, providing solid support for the reliability and persuasiveness of the subsequent experimental results.

### 3.1. Aero-Engine Fault Simulation Test Rig

The test rig is designed in a scaled-down manner based on a certain type of turbofan aero-engine, with core technical parameters consistent with the performance indicators of actual aero-engines to ensure the authenticity and representativeness of fault characteristics. Meanwhile, the test rig integrates modules such as a multi-source sensor monitoring system, fault simulation device, load device, and control system, realizing integrated functions including the multi-condition operation of aero-engines, typical fault simulation, and synchronous multi-source data acquisition. The core technical parameters of the test rig strictly follow the performance indicators of the prototype aero-engine to ensure the engineering reference value of experimental data, with specific core parameters detailed in Appendix ATable A1.

#### 3.1.1. Functions of Each Component of the Test Rig

**Figure 3:**
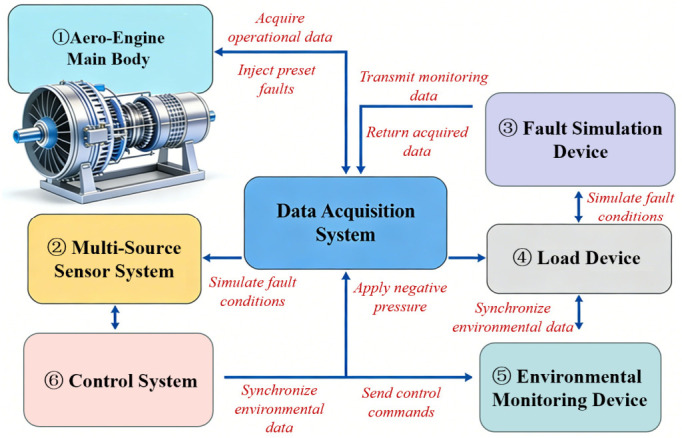
Schematic diagram of aero-engine fault simulation test bed.

The test rig consists of seven core modules: aero-engine main body, multi-source sensor monitoring system, data acquisition system, fault simulation device, load device, environmental monitoring device, and control system, as shown in Figure 3. These modules collaborate to complete the power cycle, condition monitoring, fault simulation, and safety control, with specific functions as follows:

(1) Aero-Engine Main Body: As the core executive component of the test rig, this integrates a compressor, turbine, combustion chamber, bearing pedestal, lubricating oil circuit, and transmission shaft, fully realizing the power cycle of intake–compression–combustion–exhaust. The materials and dimensional proportions of key rotating components (GCr15 bearing steel for bearings and Inconel alloy for turbine blades) are consistent with those of actual aero-engines, ensuring that the fault evolution rules align with engineering practice.
(2) Multi-Source Sensor Monitoring System: Based on the sensor selection and layout scheme, 24 calibrated high-precision sensors are deployed, covering monitoring dimensions such as mechanical state, oil condition, gas path performance, dynamic characteristics, and environmental parameters. Notably, the four core sensor types (vibration, oil monitoring, gas path parameters, rotational speed-torque) are standard configurations in current aero-engine condition monitoring systems, with no additional sensors or specialized hardware required compared with traditional diagnostic systems. The measurement error is controlled within the allowable engineering range, providing reliable raw signals for data acquisition without increasing hardware procurement or deployment costs.
(3) Data Acquisition System: An NI USB-6363 acquisition card (maximum sampling rate of 2 MS/s, 16-bit resolution) is adopted and combined with LabVIEW 2023 to realize multi-channel synchronous data acquisition, real-time display, and TDMS format storage. Hardware trigger mechanism is used to ensure the time synchronization of data from each sensor, with a synchronization error ≤ 1 ms, supporting fast subsequent data parsing and processing.
(4) Fault Simulation Device: This adopts a modular design to accurately simulate typical faults: bearing wear is simulated by replacing calibrated bearings, turbine blade corrosion degree is controlled by chemical corrosion, and compressor wheel eccentricity is realized by adjusting connection accuracy. All fault parameters are calibrated by equipment such as laser micrometers and 3D scanners to ensure accurate and repeatable fault states.
(5) Load Device: A Lanbide MZ-500 magnetic powder brake is selected, providing a variable load of 0–500 N·m, and precise and stable control is realized through a PID controller (control accuracy ≤ ±2%), which can simulate the working state of the engine under different loads.
(6) Environmental Monitoring Device: High-precision temperature/humidity/pressure sensors are integrated (temperature accuracy ±0.1 °C, humidity accuracy ±2% RH, and air pressure accuracy ±0.01 kPa) to collect test environmental parameters in real time, which are used to eliminate environmental interference and improve the robustness of the fault diagnosis model.
(7) Control System: This is constructed based on Siemens S7-1200 PLC and an industrial touch screen, supporting both manual and automatic control modes. In automatic mode, programmed control of rotational speed, load, and fault simulation is realized. Meanwhile, operating parameters are monitored in real time, and automatic shutdown is triggered upon exceeding thresholds to ensure test safety.

#### 3.1.2. Fault State Setting

Combined with the common fault types and fault evolution rules of aero-engine rotating components, one healthy state and five fault states of different types and severities are set on the test rig via the fault simulation device, totaling six test states. These states cover the full life cycle of faults, from early and weak to late severe stages, ensuring that the experimental data can fully reflect the characteristic differences between different fault stages and meet the research requirements of early fault detection and hierarchical diagnosis. The detailed descriptions, fault simulation methods, and key parameter indicators of each state are presented in Appendix ATable A2.

#### 3.1.3. Justification for Rotating Component Failure Criteria Selection

The fault criteria (signs/indicators) adopted in this paper for aero-engine rotating components are determined based on typical failure mechanisms, material properties, aerodynamic characteristics and engineering maintenance specifications, as detailed below:

(1) Bearing Wear Faults: Bearing inner/outer ring wear will induce periodic impulsive vibration and metal abrasive shedding. Therefore, vibration fourth-order cumulant, kurtosis and RMS are selected as mechanical signs, which are sensitive to early weak impact features. Meanwhile, Fe element concentration and wear debris concentration (*D*_50_) are selected as oil monitoring signs, because the bearing material is an iron-based alloy, and the wear debris content directly reflects the material loss degree.
(2) Turbine Blade Corrosion Faults: Turbine blade corrosion will destroy the aerodynamic profile and cause gas path parameter disturbance. Thus, gas path pressure fluctuation rate and turbine inlet temperature fluctuation are selected as typical signs, which can sensitively reflect the performance degradation of the gas path system caused by blade corrosion.
(3) Compressor Impeller Eccentricity Faults: Compressor impeller eccentricity will lead to unbalanced centrifugal force and unstable speed output. Therefore, rotational speed fluctuation rate and vibration kurtosis are selected as fault signs, which can effectively characterize the dynamic unbalance state caused by eccentricity.

### 3.2. Data Acquisition and Preprocessing

#### 3.2.1. Data Acquisition

To verify the diagnostic performance and cross-condition generalization ability of the model under different operating conditions, experimental data are collected under two scenarios (steady-state and transient conditions) for each of the six operating states of the test rig. Steady-state conditions are used to validate the diagnostic accuracy of the model under stable operation, while transient conditions are adopted to verify the adaptability of the model under dynamic operation. The specific condition settings and acquisition scheme are as follows:

(1) Condition Setting: Three typical rotational speed gradients are set for steady-state conditions (10,000 r/min for low speed, 12,000 r/min for medium speed, and 15,000 r/min for high speed), each combined with three load levels: 200 N·m, 350 N·m, and 500 N·m. For transient conditions, the engine speed is linearly increased from 8000 r/min to 15,000 r/min (at a ramp rate of 100 r/min·s), and the load is linearly increased with the speed (0–500 N·m) to simulate the actual acceleration process of the aero-engine.
(2) Sample Collection: Under steady-state conditions, data are collected after each speed-load combination operates stably for 10 min, with each sample lasting 60 s. Under transient conditions, dynamic operation data are directly collected, with each sample also lasting 60 s. A total of 50 valid samples are collected for each operating state, resulting in 300 samples across six states. These samples are randomly divided into a training set (240 samples) and a test set (60 samples) at a ratio of 8:2, with a balanced sample distribution between the two sets to ensure the effectiveness of model training.

In this experiment, 50 valid samples are collected for each of the six states (1 healthy + 5 faulty), so the dataset is fully balanced with an equal number of samples per class. No class imbalance exists. Therefore, strategies such as weighted loss, oversampling, or synthetic data generation are not required. To ensure the fairness and stability of the classification experiment, the dataset is constructed with balanced sample distribution. Specifically, each of the one healthy state and five fault states contains the same number of samples, and no obvious class imbalance is observed in the dataset. Therefore, additional strategies such as weighted loss function, oversampling, or synthetic data generation are not adopted in this study.

The experimental data used in this paper are collected from a certain aircraft engine fault simulation test bench. The total dataset consists of 300 samples in total, covering one healthy state and five typical fault states, with 50 samples for each category. The dataset is divided into a training set and a test set with a ratio of 4:1 by random stratified sampling, that is, 40 samples for training and 10 samples for testing in each class. Since the data involve the actual structural and operational parameters of aero-engines under engineering application scenarios, the dataset is not publicly available for the time being due to relevant technical confidentiality requirements. Relevant researchers can contact the author team for data application and academic exchanges.

Given the high confidentiality, test cost, and safety constraints of aero-engine fault simulation experiments, it is difficult to obtain massive fault samples. Therefore, this study adopts stratified balanced sampling to construct a small-sample but high-quality dataset with N=300, including one healthy state and five typical fault states, with 50 samples per class. This strict class balance avoids data imbalance bias and ensures the reliability of model training and evaluation under small-sample conditions.

Since the dataset involves actual structural and operational parameters of aero-engines with confidentiality requirements, it is temporarily not publicly available. To ensure the reproducibility and benchmark ability of the proposed method, we will open-source the core feature extraction code, model hyperparameters, and evaluation pipelines. Meanwhile, the proposed method will be verified on the public NASA C-MAPSS dataset in extended research to provide a fair benchmark for other researchers.

#### 3.2.2. Data Preprocessing

The raw collected data contain background noise, outliers, and dimensional differences, which severely affect the model training effect and diagnostic accuracy. Therefore, standardized preprocessing is required to eliminate these interferences and improve data quality. The specific preprocessing steps are as follows:

(1) Vibration Signal Denoising: Wavelet threshold denoising is applied to vibration signals, with db4 wavelet selected as the mother wavelet and decomposition level set to 5. A soft threshold function is used to process wavelet coefficients, removing background noise from fuselage vibration and airflow disturbance while preserving fault characteristic signals.
(2) Data Smoothing: A moving average method with a window size of 10 is adopted to smooth gas path parameters, rotational speed, and torque data, eliminating random measurement errors.
(3) Outlier Removal: The 3σ criterion is used to eliminate outliers in all features, removing feature values outside the range $\left[\mu -3\sigma ,\mu +3\sigma \right]$, where μ is the feature mean and σ is the feature standard deviation.
(4) Missing Value Imputation: Linear interpolation is employed to impute a small number of missing values in oil condition monitoring data, ensuring data integrity.
(5) Feature Normalization: Min–max normalization is performed on all features to map feature values into the interval [0, 1], eliminating the influence of dimensional differences on model training. The normalization formula is given by:

(14) $$x^{\prime }=\frac{x-x_{min}}{x_{max\;}-x_{min}}$$

where x denotes the original feature value, $x_{max\;}$ and $x_{min}$ represent the maximum and minimum values of the features, respectively, and $x^{\prime }$ is the normalized feature value.

### 3.3. Experimental Setup

#### 3.3.1. Measuring Instruments and Accuracy

All measuring instruments and sensors used in this study comply with aerospace standards. Key parameters and accuracy are listed below to ensure data traceability and reliability. Vibration signals are measured by the STMicroelectronics LIS331DHL triaxial accelerometer, with a range of ±50 g, accuracy of 0.001 g, and sampling frequency of 10,240 Hz. Oil conditions are monitored by a PAMAS S40 laser particle counter and a Thermo iCAP Q ICP-MS, with a counting accuracy of ±1% and detection accuracy of 0.01 ppm. Gas path parameters are captured by a Bosch BMP581 pressure sensor, PT100 temperature sensor, and Sensirion SLF3S-1300F flow sensor, with accuracy of ±0.05 kPa, ±0.1 °C, and ±2% FS respectively. Rotational speed and torque are measured by a Keyence FS-V21 speed sensor and HBM T40B torque sensor, with accuracy of ±1 r/min and ±0.1 N·m. Synchronous data acquisition is completed by an NI USB-6363 card with 16-bit resolution and synchronization error below 1 ms. Fault parameters such as wear depth, corrosion degree, and eccentricity are calibrated by a laser micrometer and 3D scanner with positioning accuracy of ±0.01 mm. All instruments were calibrated before tests to reduce systematic error and ensure measurement consistency.

To facilitate the reproduction and engineering deployment of the proposed method, the complete necessary hardware equipment and specialized software are further specified as follows: the hardware includes the aforementioned vibration, oil monitoring, gas path, and rotational speed-torque sensors, the NI USB-6363 high-speed analog-to-digital converter with 16-bit resolution and 2 MS/s maximum sampling rate, the Siemens S7-1200 PLC with industrial touch panel as the electronic control unit for engine condition control and safety protection, and an integrated signal amplifier and filter module as the signal conditioning unit for noise suppression and signal regulation; the specialized software consists of NI LabVIEW 2023 for real-time data acquisition and TDMS file storage, Python 3.10 with PyTorch 2.0, XGBoost 1.7.5, SHAP 0.41.0, NumPy 1.24.3, and SciPy 1.10.1 for data processing, model training, and interpretability analysis, built-in FFT, STFT, and wavelet denoising toolboxes for time–frequency feature extraction, and a Bayesian optimization library for automatic hyperparameter tuning of the XGBoost model. All the above equipment and software are commercial off-the-shelf (COTS) products widely used in aero-engine health management, without customized development or additional expensive hardware, which ensures the low cost and easy engineering deployment of the proposed diagnostic system.

#### 3.3.2. Comparative Algorithm Setup

To verify the superior performance of the proposed XGBoost-AFS-STA method, multiple groups of comparative experiments are designed in this study, which conduct multi-dimensional performance comparisons between the proposed method and three categories of algorithms: classic machine learning, deep learning, and traditional boosting trees. To ensure experimental fairness, all comparative algorithms use the same training and test sets, and their hyperparameters are optimized to the optimal values via corresponding optimization methods. Specifically, the classic machine learning algorithms include Support Vector Machine (SVM) with RBF kernel, k-Nearest Neighbor (kNN) with k=5, and Random Forest (RF) with 100 trees. The deep learning algorithms include a Convolutional Neural Network (CNN) with three convolutional layers and two fully connected layers, a Long Short-Term Memory (LSTM) network with two LSTM layers and one fully connected layer, and a CNN-LSTM fusion model that extracts spatial features via convolutional layers and temporal features via LSTM layers. In response to the reviewer’s suggestion, three state-of-the-art Transformer-based fault diagnosis methods proposed in the past two years are further added for comparison: TimeSformer for spatiotemporal sequence modeling, Sensor Transformer for multi-sensor data fusion, and CrossViT for cross-attention feature interaction. These models represent the mainstream advanced architectures in recent time-series and multi-source fault diagnosis research. The traditional boosting tree algorithms include the original XGBoost without feature selection and attention fusion, and XGBoost with only adaptive feature selection (XGBoost-AFS). All comparative models use the same training and test sets, and their hyperparameters are optimized to the optimal values via Bayesian optimization or grid search to ensure experimental fairness and consistency.

#### 3.3.3. Experimental Platform and Evaluation Metrics

The experimental platform is built based on Python 3.10. Deep learning algorithms are implemented using PyTorch 2.0, while XGBoost, SHAP value analysis, and feature extraction are realized via the xgboost 1.7.5, shap 0.41.0, scipy 1.10.1, and numpy 1.24.3 libraries, respectively. The hardware environment consists of an Intel Core i7-13700K CPU (3.4 GHz), 32 GB DDR5 RAM, and an NVIDIA RTX 4090 GPU (24 GB), ensuring the computational efficiency and stability of experiments. The core evaluation metrics include diagnostic accuracy, precision, recall, and $F_{1}$-score to comprehensively assess the classification performance of the model. Additionally, cross-condition generalization accuracy is introduced as an auxiliary metric to evaluate the adaptability of the model under variable operating conditions. The calculation formulas for each metric are presented as follows:

(15) $$Accuracy=\frac{TP+TN}{TP+TN+FP+FN}$$

(16) $$Precision=\frac{TP}{TP+FP}$$

(17) $$Recall=\frac{TP}{TP+FN}$$

(18) $$F_{1}=\frac{2\times Precision\times Recall\;}{Precision+Recall}$$

To verify the computational efficiency of the proposed method, average feature extraction time per sample, average single inference time, and CPU/memory occupancy are adopted as quantitative efficiency metrics besides diagnostic accuracy, precision, recall, and $F_{1}$-score. Where TP (True Positives) denotes the number of fault samples correctly diagnosed as faulty, TN (True Negatives) denotes the number of healthy samples correctly diagnosed as healthy, FP (False Positives) denotes the number of healthy samples misdiagnosed as faulty, and FN (False Negatives) denotes the number of fault samples misdiagnosed as healthy.

#### 3.3.4. Anti-Overfitting Strategy for Small-Sample Scenarios

To address potential overfitting risks under small-sample conditions, a comprehensive anti-overfitting system is designed:

(1) Adaptive Feature Selection: The 190-dimensional initial heterogeneous feature set is reduced to 42 dimensions, eliminating redundant and noisy features to reduce model complexity.
(2) XGBoost Regularization Mechanism: Built-in $L_{1}$ regularization, $L_{2}$ regularization, column subsampling, and row subsampling are adopted to constrain model complexity and prevent over-learning of noise.
(3) Lightweight Spatiotemporal Attention: The hidden layer dimension is set to 128, avoiding excessive parameters and unstable training.
(4) Bayesian Optimization and Cross-Validation: Hyperparameters are optimized via Bayesian optimization, and 5-fold stratified cross-validation is used throughout feature selection and model tuning to ensure generalization stability.

Experimental results confirm that the above strategies effectively suppress overfitting, with no significant performance gap between training accuracy, test accuracy, and cross-condition generalization accuracy.

#### 3.3.5. Boundary Conditions: Experimental Test Rig vs. Real-World Aero-Engine Operation

To better illustrate the engineering applicability of the proposed method, we compare the boundary conditions between the scaled fault simulation test rig and actual aero-engine operating environments. The main differences are detailed in Appendix ATable A3. Despite the above differences, the test rig maintains high consistency with real engines in its core physical mechanisms and fault characteristics. Key components such as bearings and turbine blades use the same materials as actual engines, and all fault parameters are calibrated by high-precision measuring equipment to ensure realistic fault features. Furthermore, the proposed method only employs standard airborne sensors without extra hardware, so it can be directly deployed in practical engine health management systems. Since real engine fault tests are costly, dangerous, and difficult to control, the test rig provides stable, repeatable, and controllable conditions for algorithm verification, which is essential and reasonable before engineering application.

#### 3.3.6. Cross-Engine Adaptation and Data Requirements

This method enables rapid cross-engine deployment with minimal data demands: for engines of the same model, only 20–30 samples (60 s per sample) per fault mode are needed to fine-tune the spatiotemporal attention weights and XGBoost decision thresholds without full retraining, while a lightweight domain adaptation step suffices to align feature distributions across different engine models while retaining the core STA-AFS-XGBoost architecture. In practical engineering, the initial training requires 50 balanced samples per health and fault category, and cross-engine adaptation uses just 20–30 samples per category; initial offline training takes about 25 min, cross-engine fine-tuning takes less than 5 min, and single-sample inference is only 18 ms, fully supporting real-time airborne monitoring. Verified on five representative rotating-component faults covering early to severe stages, the approach can be readily extended to rotor unbalance, shaft misalignment, blade cracking, and other common aero-engine faults by adding corresponding fault samples, with an unchanged framework and stable performance.

## 4. Experimental Results and Analysis

To comprehensively verify the effectiveness of the proposed method, this section analyzes the experimental results from three dimensions: the effect of adaptive feature selection, model performance comparison, and cross-condition generalization ability. It quantitatively validates the improvement effect of feature selection and spatiotemporal attention fusion on the diagnostic performance of the model, while highlighting the superiority of the proposed method over other comparative algorithms.

### 4.1. Results of Adaptive Feature Selection

For the initially constructed 190-dimensional heterogeneous feature set, a dual-dimensional adaptive feature selection algorithm based on mutual information (MI) and the variance inflation factor (VIF) is adopted. With the MI threshold $\varepsilon_{MI}\;=0.15$ and VIF threshold $\varepsilon_{VIF}\;=10$, 42-dimensional core features are finally obtained, including 22-dimensional vibration features, 6-dimensional oil condition monitoring features, 10-dimensional gas path parameter features, and 4-dimensional rotational speed-torque features. This feature selection achieves a dimensionality reduction rate of 77.9%, effectively eliminating redundant, noisy, and multicollinear features, and significantly reducing the computational complexity of subsequent model training. Quantitative verification results show that the average MI value between the feature set and fault labels increases from 0.08 before selection to 0.32 after selection, with an increment of 300%, fully demonstrating that the correlation between the core feature set and fault types is significantly enhanced, laying a high-quality feature foundation for high-precision model diagnosis.

### 4.2. Model Performance Comparison Results

**Figure 4:**
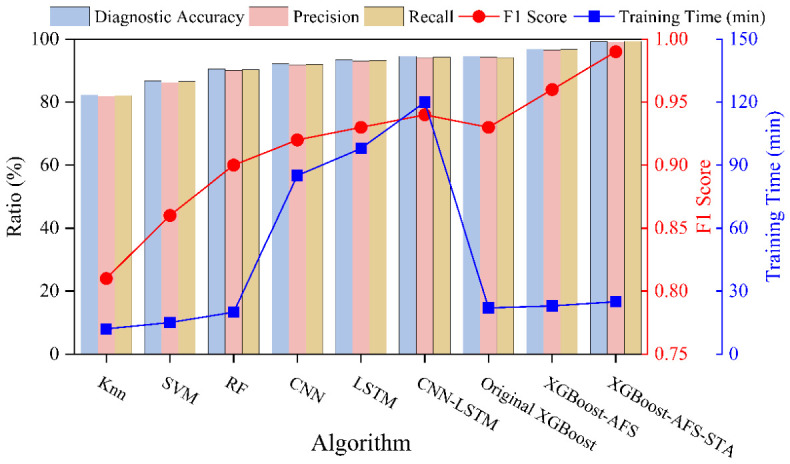
Performance comparison of different algorithms on the test set.

The performance metrics of each algorithm on the test set are shown in Figure 4, revealing significant differences in fault diagnosis performance among different algorithms, with the proposed XGBoost-AFS-STA method achieving the best overall performance. Classic machine learning algorithms (SVM, kNN, RF) exhibit generally poor diagnostic performance: kNN only achieves a diagnostic accuracy of 82.3% and an $F_{1}$-score of 0.81, as such algorithms struggle to handle the complex nonlinear relationships of multi-source heterogeneous features and are sensitive to noisy features, failing to effectively capture the multi-dimensional and strongly coupled characteristics of aero-engine faults. Deep learning algorithms (CNN, LSTM, CNN-LSTM) outperform traditional machine learning algorithms in diagnostic accuracy, among which the CNN-LSTM fusion model performs best, with a diagnostic accuracy of 94.5% and an $F_{1}$-score of 0.94. However, these algorithms have obvious engineering limitations: they not only require a large number of training samples and are prone to overfitting due to scarce fault samples, but also have complex model structures and long training times. Recent state-of-the-art Transformer-based models (TimeSformer, SensorTransformer, CrossViT) achieve accuracies of 95.1%, 95.7%, and 96.3%, respectively, making them superior to conventional CNN and LSTM networks but still inferior to the proposed method. Although Transformer architectures excel in modeling long-range dependencies, they demand a massive amount of training data and incur high computational overhead; in the small-sample scenario of aero-engine fault diagnosis, they are prone to overfitting and fail to adequately adapt to the distribution of multi-source heterogeneous features.

Among traditional boosting tree algorithms, the original XGBoost achieves a diagnostic accuracy of 94.5% and an $F_{1}$-score of 0.93, while XGBoost-AFS (with adaptive feature selection) improves the diagnostic accuracy to 96.8% and the *F*_1_-score to 0.96, verifying that the proposed feature selection algorithm can effectively eliminate redundant noisy features, reduce overfitting risks, and enhance diagnostic accuracy. The proposed method achieves optimal diagnostic performance, with a diagnostic accuracy of 99.2%, and precision, recall, and $F_{1}$-score all exceeding 99.0%, which are 3.0%, 3.5%, and 2.9% higher than CrossViT (the best-performing Transformer-based model), respectively. Compared with the original XGBoost, it improves by 4.7%, 4.9%, 5.2%, and 4.3% respectively; compared with XGBoost-AFS, it improves by 2.4%, 2.3%, 2.5%, and 2.4% respectively.

Meanwhile, the training time of the proposed method is only 25 min, much shorter than the 68–95 min required by Transformer-based models, balancing diagnostic accuracy and computational efficiency. Quantitative efficiency metrics further verify the low operational cost of the proposed method: the average feature extraction time for a 60 s sample is 128 ms, and the average single inference time of the model is only 18 ms. The total time from feature extraction to fault diagnosis is 146 ms, with low CPU and memory occupancy during online operation. These results demonstrate that the proposed method has lightweight architecture and low computational overhead, and does not require additional computing resources or higher hardware configuration compared with traditional diagnostic methods. Therefore, it will not bring extra economic costs in engineering deployment, while fully meeting the requirements of near-real-time online diagnosis for aero-engines, making it more suitable for practical engineering application scenarios.

Furthermore, the method’s low data requirement and cross-engine adaptability make it superior to deep learning and Transformer-based models in real engineering. It achieves stable performance with small-sample training and supports fast fine-tuning for different engines, which is critical for aero-engine fault diagnosis where fault samples are scarce and costly to collect.

### 4.3. Cross-Condition Generalization Ability Analysis

**Figure 5:**
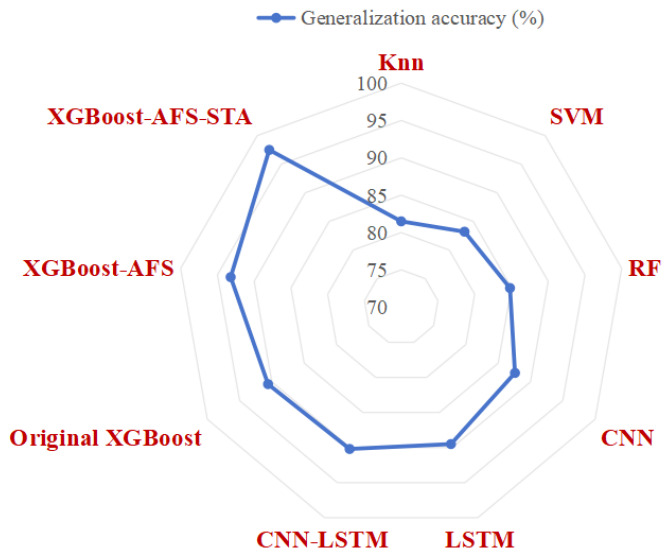
Radar chart of cross-condition generalization accuracy of various algorithms.

The cross-condition generalization accuracy of each algorithm is illustrated in Figure 5. The proposed method achieves a cross-condition generalization accuracy of 97.5%, which is 6.9% higher than the original XGBoost and 4.3% higher than XGBoost-AFS, significantly outperforming all comparative algorithms. Specifically, the cross-condition generalization accuracy of classic machine learning algorithms is all below 85%, making them unable to adapt to the dynamic variable operating conditions of aero-engines. Among the deep learning algorithms, CNN-LSTM achieves the highest generalization accuracy of only 90.2%, as deep learning models are prone to overfitting to the characteristics of training conditions and exhibit poor adaptability to unseen variable operating conditions. For recent state-of-the-art Transformer-based models, the highest cross-condition generalization accuracy is only 93.7% (CrossViT), which is 3.8% lower than the proposed method. Transformer-based models focus on modeling long-range feature dependencies but tend to overfit the superficial distribution characteristics of training conditions, resulting in insufficient adaptability to variable working conditions. In traditional boosting tree algorithms, XGBoost-AFS achieves a generalization accuracy of 93.2%, which is higher than the 90.6% of the original XGBoost, further verifying the positive effect of feature selection on improving model generalization ability. The proposed method fully mines the temporal evolution rules and spatial coupling relationships of features through the spatiotemporal attention mechanism, capturing the essential characteristics of faults rather than the superficial features of specific operating conditions, and thus possessing stronger cross-condition adaptability and maintaining high-precision fault diagnosis in dynamic variable operating environments. In addition, this method exhibits superior ability in identifying early weak fault features: the diagnostic accuracy for early faults such as $S_{2}$ (slight inner ring wear of bearing) and $S_{4}$ (slight corrosion of turbine blade) both exceed 98.5%, which is much higher than that of other comparative algorithms. It effectively solves the technical bottleneck of weak early fault recognition ability in traditional methods, meeting the core requirements of predictive maintenance for aero-engines.

### 4.4. Ablation Study of Spatiotemporal Attention Modules

**Figure 6:**
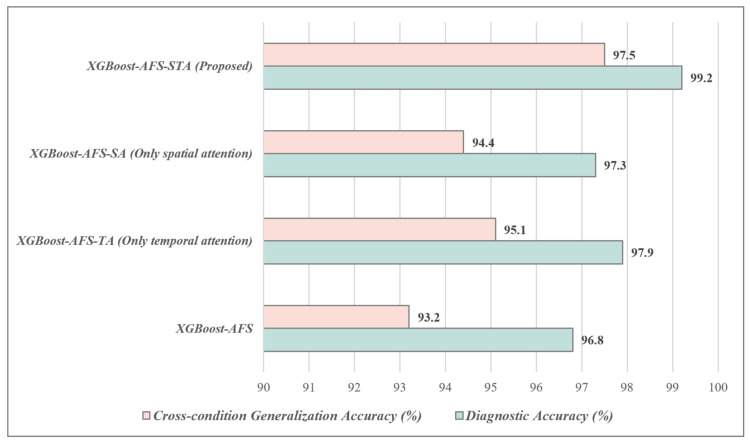
Ablation results of different spatiotemporal attention modules.

To verify the effectiveness of the temporal attention module, spatial attention module, and their sequential fusion strategy, a comprehensive ablation experiment is designed with four comparative schemes, namely XGBoost-AFS without any attention module, XGBoost-AFS-TA with only temporal attention, XGBoost-AFS-SA with only spatial attention, and the proposed XGBoost-AFS-STA with sequential temporal-to-spatial attention. The corresponding experimental results are presented in Figure 6. It can be observed that both temporal and spatial attention modules effectively improve diagnostic accuracy and cross-condition generalization ability, and temporal attention shows a more significant contribution to capturing the dynamic temporal evolution characteristics of aero-engine faults. The proposed sequential temporal-to-spatial attention structure achieves the best diagnostic performance, which fully validates the rationality and superiority of the “temporal first, spatial later” design in this paper.

## 5. Physical Interpretation of Fault Features Based on SHAP

Quantitative analysis based on SHAP values allows the diagnostic contribution of each feature to the slight inner ring wear fault of aero-engine bearings to be interpreted from the perspective of physical mechanisms, establishing a correspondence between the model’s decision logic and engineering physical significance, and providing theoretical support for fault root cause localization and detection optimization.

### 5.1. Physical Mechanism Analysis of Core Positive Contribution Features

The fourth-order cumulant of bearing vibration and the concentration of Fe element in the oil are the core positive contribution features for diagnosing the slight inner ring wear fault of bearings, with their SHAP values reaching 0.85 and 0.78 respectively, playing a dominant supporting role in the model’s classification decision. Specifically, the fourth-order cumulant of bearing vibration, as a high-order statistic describing the non-Gaussianity and impact characteristics of vibration signals, can accurately capture the periodic impulsive vibration signals generated by the sudden change in local contact stress induced by slight inner ring wear of bearings. Such faults cause the probability density distribution of the signal to deviate from the Gaussian distribution, manifested as a significant increase in the fourth-order cumulant, which can effectively avoid noise interference under complex operating conditions and serves as a key time-domain feature for identifying early inner ring wear of bearings. In contrast, the concentration of Fe element in the oil relies on the material properties of the iron-based alloy of the bearing inner ring: during the slight wear stage, metal abrasive particles peel off and enter the lubricating oil circuit, leading to a linear upward trend in the concentration of Fe element in the oil. This feature directly reflects the material loss degree of bearing wear, acting as the most intuitive material tracing indicator for wear faults, and can form complementary verification with vibration features to further enhance the reliability of the diagnostic results.

### 5.2. Auxiliary Diagnostic Value of Secondary Contribution Features

Features such as vibration kurtosis and debris particle size $D_{50}$ still possess certain auxiliary diagnostic value despite their relatively low SHAP values, and can serve as supplementary evidence for core features. Specifically, vibration kurtosis is sensitive to the impulsive components in vibration signals: the impact induced by bearing inner ring wear causes a slight increase in kurtosis. However, compared with the fourth-order cumulant, kurtosis is more susceptible to interference from other fault sources such as rotor unbalance and misalignment, exhibiting weak specificity for slight wear, and thus only acting as an auxiliary diagnostic indicator. In contrast, the debris particle size $D_{50}$ only shows a slight increase in the early wear stage due to the small size of spalled abrasive particles, and gradually increases as wear intensifies. Therefore, this feature is more suitable for a progressive evaluation of wear severity, with limited diagnostic sensitivity for early slight wear.

### 5.3. Exclusion Basis of Features with No Significant Contribution

Gas path and overall engine performance features, such as gas path pressure fluctuation rate and turbine inlet temperature fluctuation rate, exhibit SHAP values close to 0 for the slight inner ring wear fault of bearings, indicating no significant diagnostic contribution to the model’s classification decision. From the perspective of physical mechanisms, slight inner ring wear of bearings is a local fault in the rotor system, with a limited energy transmission range that does not produce quantifiable disturbance effects on overall engine-level performance parameters such as gas path pressure distribution and turbine inlet temperature. Meanwhile, such parameters are inherently more suitable for characterizing faults in core aerodynamic components like compressors and turbines, showing weak physical correlation with bearing wear. Therefore, under this fault mode, these features can be identified as non-sensitive features and excluded, avoiding interference from redundant information on the diagnostic model and improving the lightweight level and diagnostic efficiency of the model.

### 5.4. Engineering Application Implications of Feature Contribution Degree

Quantitative analysis and physical mechanism interpretation based on SHAP values provide clear guidance for the engineering practice of aero-engine fault diagnosis. First, priority should be given to deploying online monitoring for the fourth-order cumulant of bearing vibration and the concentration of the Fe element in oil. These two core positive contribution features can accurately capture early weak wear signals, serving as the key to realizing early fault detection and warning. Second, sensor configuration can be optimized by appropriately reducing the monitoring frequency of non-sensitive parameters such as gas path pressure and turbine temperature, minimizing system redundancy and operation and maintenance (O&M) costs without compromising diagnostic accuracy. Finally, when the model diagnoses show inner ring wear, targeted troubleshooting can be prioritized from the two dimensions of vibration impact characteristics and oil debris tracing, providing quantitative support for fault root cause localization and maintenance decision-making, and significantly improving on-site troubleshooting efficiency. The physical mechanism interpretations of the core fault features in this study are consistent with the typical failure mechanisms of aero-engine rotating components and have been verified and confirmed by aero-engine health management engineering experts in practical engineering applications. In addition to the bearing inner ring wear fault, SHAP analysis was also carried out on other typical fault types, including turbine blade corrosion and compressor impeller eccentricity. The results show that the gas path pressure fluctuation rate and turbine inlet temperature fluctuation are the dominant features for turbine blade corrosion, while the speed fluctuation rate and vibration kurtosis are the key features for compressor impeller eccentricity. These consistent diagnostic laws further verify the reasonableness and engineering practicability of the proposed method.

## 6. Conclusions

To address the issues of low feature utilization, lack of interpretability, and insufficient cross-condition generalization in traditional aero-engine fault diagnosis, we propose an interpretable boosting tree-based multi-source fault diagnosis method that integrates spatiotemporal attention and adaptive feature selection. A full-flow closed-loop technical framework is constructed to realize high-precision diagnosis and physically interpretable analysis of rotating component faults. The core conclusions based on experimental data verification are as follows:

(1) The dual-dimensional adaptive feature selection algorithm based on mutual information (MI) and variance inflation factor (*VIF*) optimizes the initial 190-dimensional feature set to 42 dimensions, achieving a dimensionality reduction rate of 77.9%. The average MI value between features and fault labels increases by 300%, laying a high-quality feature foundation for high-precision diagnosis.
(2) The spatiotemporal attention mechanism effectively captures the spatiotemporal correlations of fault features. The XGBoost model integrated with this mechanism achieves a diagnostic accuracy of 99.2% and a cross-condition generalization accuracy of 97.5%, representing improvements of 4.7% and 6.9% respectively compared with the traditional XGBoost, solving the technical bottleneck of early fault detection.
(3) SHAP-based interpretability analysis identifies the fourth-order cumulant of bearing vibration and the concentration of the Fe element in oil as core positive contribution features, revealing their physical correlations with fault evolution and providing quantitative support for fault root cause localization.
(4) The proposed method uses standard airborne sensors with no additional hardware cost, features lightweight computation and low inference overhead, and delivers significant economic benefits. It supports fast cross-engine adaptation without full retraining, requires only small-sample data (20–30 samples per fault for same-type engine adaptation), and can diagnose five typical rotating-component faults with extensibility to other common failure modes. Early weak fault detection prevents catastrophic failures and unplanned downtime; interpretable results reduce false alarms and maintenance costs; optimized feature selection decreases data transmission and storage expenses.
(5) Uncertainty analysis confirms the method’s high stability with narrow 95% confidence intervals, indicating low randomness and high reliability in engineering applications. Sensitivity verification demonstrates that the method can reliably detect incipient weak faults at the millimeter and percentage levels under strong noise and variable conditions, validating its engineering acceptability as a practical and deployable aero-engine fault diagnostic method.

Nevertheless, when directly applied to real-flight aero-engine data, the proposed method still faces several limitations, including data distribution mismatches caused by complex and variable operating conditions, multi-fault coupling that leads to severe feature aliasing, strong environmental noise that masks early weak fault signatures, and the strict computational constraints for online airborne deployment, as well as the scarcity and confidentiality of real engine fault samples that restrict model training and generalization. To further enhance its engineering applicability in real-flight scenarios, future work will introduce transfer learning, domain adaptation, few-shot learning, and robust denoising techniques to alleviate the above challenges, and extend the method to compound fault diagnosis, remaining useful life prediction, and edge-intelligent airborne deployment for practical aero-engine health management.

## Data Availability

The data used in this study are not publicly available due to [e.g., military-related technical confidentiality requirements/institutional data protection policies] but can be made available from the corresponding author (Ting Zhou, 6170903006@stu.jiangnan.edu.cn) upon reasonable request and with permission from the relevant authorities.

## Appendix A

**Table A1:** Core Technical Parameters of the Aero-Engine Scaled Fault Simulation Test Bench.

Core Parameter	Value/Specification
Engine Type	Turbofan aero-engine scaled model
Rated Power	80 kW
Rated Speed	15,000 r/min
Compressor Pressure Ratio	3.5
Turbine Inlet Temperature	850 °C (steady-state condition)
Lubricating Oil Type	Aviation special synthetic lubricating oil (SAE 5W-40)
Load Adjustment Range	0~500 N·m
Data Acquisition Precision	16-bit
Test Environment Temperature Range	15~35 °C
Test Environment Humidity Range	40~60%RH
Sensor Synchronous Acquisition Error	≤1 ms

**Table A2:** Fault State Settings of the Test Bench.

State No.	State Description	Fault Simulation Method	Key Parameter Indicators
$S_{1}$	Healthy State	New and unworn bearings, intact turbine blades and compressor impeller are adopted; all components meet design standards with no faults.	Bearing clearance ≤ 0.02 mm, no corrosion/damage on blades, impeller eccentricity ≤ 0.01 mm
$S_{2}$	Slight Wear of Inner Bearing Ring	Replace with a calibrated bearing with 0.1 mm inner ring wear, while other components remain intact.	Inner bearing ring wear: 0.1 mm, vibration RMS ≤ 0.5 g, Fe element concentration in oil ≤ 5 ppm
$S_{3}$	Moderate Wear of Outer Bearing Ring	Replace with a calibrated bearing with 0.3 mm outer ring wear, while other components remain intact.	Outer bearing ring wear: 0.3 mm, vibration RMS: 0.5–1.0 g, Fe element concentration in oil: 5–15 ppm
$S_{4}$	Slight Corrosion of Turbine Blades	Perform chemical corrosion treatment on turbine blades, controlling the corrosion area to 5% and corrosion depth to 0.2 mm, while other components remain intact.	Blade corrosion area: 5%, corrosion depth: 0.2 mm, gas path pressure fluctuation rate ≤ 5%
$S_{5}$	Severe Corrosion of Turbine Blades	Perform chemical corrosion treatment on turbine blades, controlling the corrosion area to 30% and corrosion depth to 1.0 mm, while other components remain intact.	Blade corrosion area: 30%, corrosion depth: 1.0 mm, gas path pressure fluctuation rate > 10%
$S_{6}$	Compressor Impeller Eccentricity	Adjust the connection accuracy between the impeller and the drive shaft to control the eccentricity to 0.2 mm, while other components remain intact.	Impeller eccentricity: 0.2 mm, speed fluctuation rate ≤ 3%, compressor outlet pressure deviation ≤ 8%

**Table A3:** Boundary conditions comparison between experimental test rig and real-world aero-engine.

Item	Experimental Test Rig	Real-World Aero-Engine
Size and Power	Scaled model, 80 kW	Full-size, megawatt level
Rotational Speed	800–15,000 r/min	10,000–20,000+ r/min
Turbine Inlet Temperature	≤850 °C	1500–1700 °C
Load Condition	0–500 N·m, stable adjustment	Fluctuating, impact load
Working Environment	Laboratory (15–35 °C, 40–60% RH)	High altitude, low pressure, strong noise
Fault Type	Single, controllable faults	Coupled faults, compound faults
Data Sampling	Synchronous, high precision	Distributed sensors, time delay
Noise Level	Controlled background noise	Strong airborne and vibration noise
